# Burgeoning burden of non-communicable diseases in Nepal: a scoping review

**DOI:** 10.1186/s12992-015-0119-7

**Published:** 2015-07-16

**Authors:** Shiva Raj Mishra, Dinesh Neupane, Parash Mani Bhandari, Vishnu Khanal, Per Kallestrup

**Affiliations:** Nepal Development Society (NEDS), Bharatpur-10, Chitwan, Nepal; School of Population Health, University of Western Australia, Crawley, Australia; Center for Global Health, Department of Public Health, Aarhus University, Aarhus, Denmark; Institute of Medicine, Maharajgunj Medical Campus, Kathmandu, Nepal; School of Public Health, Curtin University, Bentley, Australia

**Keywords:** Non communicable diseases, Prevalence, Review, Nepal

## Abstract

In the last decades, prevalence of non-communicable diseases (NCDs) has escalated in Nepal. This study reviews existing evidence on the burden of non-communicable diseases in Nepal using the framework developed by Arksey and O’Malley for scoping reviews. A total of 110 articles were identified from database searches, and four from additional searches. The titles and abstracts were reviewed using predetermined screening criteria. We limited our search to existing literature in English language and included all studies regardless of year of study. Both observational and interventional studies were included. Studies conducted outside Nepal and studies not reporting prevalence of NCDs were excluded. Additionally, we searched reference lists of included publications. All previous reports of Step Wise Surveillance to NCDs (STEPS Surveys) were included in the review. Finally, a total of 60 articles were included in this review. Limited studies on population-based prevalence of mental illness, chronic respiratory diseases, cardiovascular diseases, and road traffic accidents were found. There were limitations in the studies related to generalizability due to small sample sizes, non-random sampling and lack of studies from certain region of country. Nevertheless, high prevalence of hypertension and diabetes was found. Similarly, hospital-based studies reported high burden of cardiovascular diseases among outpatient contacts. Population-based cancer registries do not exist in Nepal. However, existing studies report 8,000-10,000 cancer deaths annually in Nepal. The most common cancer site in males was the lung, followed by the oral cavity and gastric, while the first three in females were cervix uteri, breast and lung. Prevalence of psychiatric morbidity was also high. Despite alarming burden of NCDs, the country’s response is weak. Nepal needs to build non-communicable disease programmes with focus on disease prevention and management as well as awareness activities in urban and rural settings at community level.

**Electronic supplementary material:**

The online version of this article (doi:10.1186/s12992-015-0119-7) contains supplementary material, which is available to authorized users.

## Background

Globally, non-communicable diseases (NCDs) accounted for 68 % of all deaths in 2012; 74 % of these deaths occurred in low- and middle-income countries (LMIC) [1]. Epidemiological transition of disease in LMIC, communicable and non-communicable, and demographic changes with an ageing population is changing health priorities in LMICs. Along with globalization, risk factors of NCDs have also been globalized. Globalization and urbanization has fostered the rate of physical inactivity [2, 3], increased the marketing of tobacco and alcohol [4] and has changed the food cultures, consumption and dietary pattern [5]. In addition to that, wide disparity in access to health services and increase in out-of-pocket expenditure to health care is increasing in LMIC [6], with Nepal being no exception.

Nepal, a small Himalayan country, had a high prevalence of communicable diseases a few decades ago; now the country has higher age-standardized death rates and disability-adjusted life years from NCDs than communicable diseases (CDs) [7]. In Nepal, NCDs account for more than 44 % of deaths and 80 % of outpatient contacts. Nearly one third of the population has hypertension [8] and 15 % has diabetes [9]. Chronic obstructive pulmonary diseases (43 %) are the most common NCDs among outpatients followed by cardiovascular disease (40 %), diabetes mellitus (12 %) and cancer (5 %) [10]. Furthermore, earlier studies have reported a higher level of alcohol and tobacco use in Nepal [11–13]. Rapid urbanization, change in dietary patterns, behavioural factors and major improvements in prevention of maternal and child health to raise life expectancy are all factors contributing to shift disease patterns in Nepal [14].

Recently, Nepal has taken steps in the control of NCDs through the ratification of a national policy in 2009, and a strategy and plan of action in 2014 for prevention and control of NCDs [15, 16]. Implementing such policies into practice requires knowledge of the burden of NCDs and a national registry system to monitor NCDs does not exist in Nepal. Moreover, there are no available nationwide prevalence studies on NCDs. Thus, documentation of available evidence from existing studies will serve as an information base which the policy makers and program planners can use in decision making and program planning. This study reviews the burden of NCDs in Nepal.

## Methods

A scoping review was conducted using the Arksey and O’Malley framework [17]. A review framework was prepared by a team of experts to develop the overall study protocol, including identification of search terms and databases. The review included the following five key phases: (1) identifying the research question, (2) identifying relevant studies, (3) selecting studies, (4) charting of data, and (5) collating, summarizing, and reporting results [18].

**Fig. 1:**
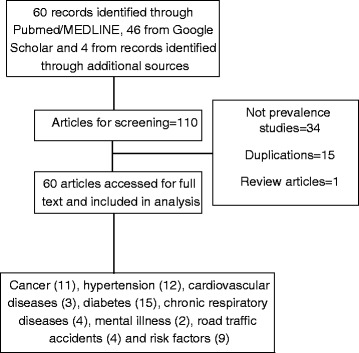
Flow chart of identified studies

A search was conducted primarily on online Medical Literature Analysis and Retrieval System (MEDLINE) using a combination of medical subject headings (MeSH); ‘Nepal’ as MeSH major topic and ‘prevalence studies’ and individual diseases and risk factors as subject headings. The search terms for the review included a combination of: ‘Nepal’; ‘noncommunicable diseases’; ‘non-communicable diseases’; ‘hypertension’, ‘diabetes’; ‘hyperglycemia’; ‘obesity’; ‘overweight’; ‘cancer’, ‘mental illness’; ‘depression’; ‘kidney’; ‘renal’; ‘liver’; ‘trauma’; ‘injuries’; ‘injury’; ‘risk factors’; ‘tobacco’; ‘smoking’; ‘alcohol’; ‘physical activity’; ‘vegetable’. The search terms used in this study is given in Additional file 1. This framework was adapted from an earlier study in Uganda [19]. Additional searches were done in Google Scholar. We obtained 110 papers: cancer/neoplasms (16), hypertension (21), cardio- vascular diseases (3), diabetes/hyperglycemia (31), chronic respiratory diseases (4), mental illness (3), road traffic accidents (RTA)/wound injuries (7) and risk factors (25). The titles and abstracts were reviewed using predetermined screening criteria. We limited our search to existing literature in English language and included all studies regardless of year of study. Both observational and interventional studies were included. Studies conducted outside Nepal, studies not reporting prevalence of NCDs were excluded. Additionally, we searched reference lists of included publications. All previous reports of Step Wise Surveillance to NCDs (STEPS Surveys) were included in the review [11–13]. Finally, a total of 60 articles were included in this review: cancer (11), hypertension (12), cardiovascular diseases (3), diabetes (15), chronic respiratory diseases (4), mental illness (2), road traffic accidents (4) and risk factors (9). Limited studies on population-based prevalence of mental illness, chronic respiratory diseases, cardiovascular diseases, and road traffic accidents were found. The studies had limitations including difficulties in generalizing on the basis of small sample studies, non-random sampling and lack geographical representativeness of the studies. A detailed flow chart of the studies is shown in Fig. 1.

The findings have been divided into eight sections. Cardiovascular diseases, chronic respiratory diseases, mental diseases, and risk factors of non-communicable diseases are presented separately in a single section.

## Results

### Cancer

The exact prevalence of cancer in Nepal is unknown due to the lack of a population-based cancer registry system [20]. Based on data from major hospitals, there are approximately 8,000-10,000 new cancer patients annually in Nepal [20]. Out of nine studies included in this review, seven were hospital-based studies describing the demographic characteristics of patients with cancer whereas two studies estimated prevalence of cancers related to the Human Papilloma Virus (HPV) at community level. Studies reported between 25 and 58 types of cancers [21, 22]. Overall, the most common cancer site in males is the lung, followed by the oral cavity and gastric in females the most common sites are cervix uteri, breast and lung [23]. The top five malignancies in Nepal are gastric, ovarian, Non-Hodgkin’s Lymphoma (NHL), lungs and breast cancer [24]. In male patients, respiratory and digestive cancers are the most prevalent with 50.5 % and 30.5 %, respectively. Among the respiratory cancer cases, lung cancer constitute 60.4 % of cases. Among female patients, reproductive and respiratory cancers are most prevalent with 62.0 % and 14.4 % of all female cancers. Earlier studies have reported a prevalence of HPV at 8.6 % [25] and 9.6 % among urban and rural undiagnosed populations, respectively [26]. A 10-year retrospective study on cervical carcinoma found that squamous cell carcinoma comprised 40 % of cases, adenocarcinoma 4 %, and both 1.1 % of cases [27]. Females are more frequently affected by cancer than males [28]. However, age-standardized annual gastric cancer has a prevalence of 3.3 in 100,000 [29] and a male-female ratio of 2.8:1 [23]. Women of advanced age have high prevalence of breast lumps and breast cancer [28, 30]. Age-wise distribution of cancer is distinct, for instance, among males, leukemia and lymphomas are high among the young followed by lung, oral, gastric, and lung cancer in middle and advanced age. Similarly, among females, breast cancer is more common in young women, whereas cervical cancer is common in middle aged and followed by lung cancer among the older age group (>60 years) [23]. (Table 1) The age group 55- 65 years accounts for about 29.0 % of total cancer cases while the 45-55 year-olds constitute 23.7 %. The incidence of cancer was found to be less in children below 15 years of age at 1.9 % [24]. There was also variation in reported cancer prevalence between hospitals, but this appeared largely because of therapeutic modalities available in different institutions [21] (Table 1).

**Table 1:** Cancers listed according to frequency in different age groups. Adapted from Multi-institution hospital-based cancer incidence data for Nepal - an initial report [21]

	0-14 years		15-34 years		35-64 years		>64 years	
	Male	Female	Male	Female	Male	Female	Male	Female
1^st^	LL (23.7 %)	Eye(27.3 %)	ML (15.1 %)	Breast(18.1 %)	Lung(26.1 %)	Cervix(26.0 %)	Lung(26.1 %)	Lung(23.2 %)
2^nd^	ML(11.8 %)	LL(14.5 %)	NHL(8.0 %)	Ovary(10.9 %)	Oral(9.3 %)	Breast(18.4 %)	Gastric(9.3 %)	CX(14.5 %)
3^rd^	Brain (11.8 %)	Bone(9.1 %)	Bone(7.1 %)	Cervix(8.5 %)	Gastric(7.4 %)	Lung(9.3 %)	Larynx(8.3 %)	Breast(6.6 %)
4^th^	Eye(10.5 %)	ML(7.3 %)	Oral (6.6 %)	ML(8.1 %)	Larynx(7.0 %)	Ovary(6.3 %)	Eso(5.4 %)	GB(6.3 %)
5^th^	HL(9.2 %)	Brain(7.3 %)	Gastric(5.7 %)	Brain(4.0 %)	Phar(4.4 %)	GB(5.5 %)	Oral(5.3 %)	Ovary(4.7 %)
6^th^	NHL(7.9 %)	LEU(7.3 %)	Rectal(5.2 %)	Rectal(4.0 %)	NHL(4.3 %)	Gastric (4.6 %)	UB(4.7 %)	Gastric(4.0 %)
7^th^	Kidney(5.3 %)	NHL(5.5 %)	Brain(4.2 %)	NHL(2.6 %)	Eso(3.6 %)	Oral(2.4 %)	Pharynx(5.4 %)	Eso(4.0 %)
8^th^	Bone(5.3 %)	HL(3.6 %)	LL(3.8 %)	URT(2.6 %)	UB(3.3 %)	NHL(1.7 %)	Prostate(3.6 %)	Oral(3.0 %)

*GB* gallbladder, *LEU* leukemia, *LL* lymphoid leukemia, *ML* myeloid leukemia, *NHL* non-Hodgkins lymphoma, *UB* urinary bladder, *Oral* Oral cavity, *Phar* Phalangeal, *Eso* Esophagus, *HL* Hodgkins Lymphoma, *CX* Cervical Cancer, *URT* Upper Respiratory Track

### Hypertension

An individual with hypertension (HTN) has a systolic blood pressure SBP > 140 mm Hg and/or diastolic blood pressure DBP > 90 mm Hg, takes antihypertensive drugs, or has previously been diagnosed with hypertension by health care workers [31]. The reported prevalence of hypertension ranges between 22.4 % and 38.6 % [32–37]. Vaidya et al. reported there has been a three-fold increment in the prevalence of HTN in the Nepalese community over the last 25 years [33]. Among suburban and urban populations 28.9 % [38] and 22.7 % [39], respectively has HTN. A study conducted among women in rural Nepal found a 3.3 % prevalence of HTN [40]. Among special groups like war veterans [41] and elderly (= > 50 years) [42] 22.7 % and 44.9 %, respectively reported HTN . Hypertension was more frequent in males (n = 2) [35, 40], increased with age (n = 4) [34, 35, 40, 43], body mass index above (n = 2) [33, 35], smoking (n = 3) [34, 35, 43], alcohol (n = 3) [34, 35, 43] and low socio-economic status (n = 2) [34, 43].

### Cardiovascular disease

Only two studies have reported the prevalence of cardiovascular disease (CVD), and one of these has estimated the prevalence based on a projection. Vaidya et al. reported the prevalence of CVD to be 5.7 % in Nepal [44]. Also, coronary artery disease was estimated to be 5 % in 2003 by Maskey et al [45]. An earlier study reported a hospital-based CVD prevalence of 40 % in outpatients. CVD was common in young age and reported as HTN (47 %), cerebrovascular accidents (16 %), congestive cardiac failure (11 %), ischemic heart disease (7 %), rheumatic heart disease (5 %) and myocardial infarction (2 %) by a study in 2009 [10].

### Diabetes

Six studies with sample sizes ranging from 920 to 17,082 have reported prevalence of diabetes between 4.1 % and 9.5 % [9, 32, 46–50]. Five studies were based on WHO criteria from 1998 [51], one was based on 1985 WHO criteria [52], and for one study, classification criteria could not be obtained. A nationwide survey of people ≥ 20 years reported prevalence of diabetes of 14.6 % in urban areas and 2.5 % in rural areas [9]. A study performed in Eastern Nepal showed a prevalence of gestational diabetes of 8.3 % [53]. A significantly higher prevalence of type 2 diabetes was reported among males compared to females [47–49]. An age-gradient in diabetes prevalence was seen as type 2 diabetes and Impaired Fasting Glucose prevalence increased with age [49, 54]. Regarding the rural-urban differences, an earlier study found high increment in diabetes prevalence over a decade in both rural (0.3 % to 2.5 %) and urban areas (1.4 % to 14.6 %) of Nepal [55]. Other studies have reported a diabetes prevalence of 19.0 % in rural [39] and 23.5 % in urban area [56] 14.9 % of cases were women [57].

Among population subgroups, the highest prevalence of diabetes was seen among the elderly in Kathmandu valley, where 25.9 % are elderly; among these elderly, 17.3 % were newly diagnosed and 8.6 % took diabetes medication [58]. Higher body mass index and HTN were significantly associated with higher prevalence of diabetes [54].

### Chronic respiratory diseases

Very few studies have been conducted on chronic respiratory diseases in Nepal. Hospital-based prevalence of respiratory diseases was reported 31.7 % in a study in 2010 and Chronic Obstructive Pulmonary Disease (COPD) (23.2 %) was the most common diagnosis [59]. Chronic bronchitis had a prevalence of 18.3 % [60]. Higher prevalence of COPD was seen after middle-age, with the highest prevalence at 60-69 years. The majority of COPD patients were women (60 %) of higher ethnic groups, and prevalence was higher among those aged 60–69 [61, 62].

### Mental illness

Only two studies have reported prevalence of psychiatric illness in Nepal, both based on the General Health Questionnaire 12 (GHQ-12) [63] and the ICD 10 classification [64]. Prevalence of mental illness was 37.5% [63]. Dissociative/conversion disorders were most common (17.2 %) among the psychiatric morbidities followed by disorders related to alcohol abuse (16.5 %) and depressive disorder (13.2 %). In addition to that 17.0 % were reported to have medical complications following attempts of suicide [64]. Mental illnesses were more common among adult males above 30 years and in disadvantaged groups such as *Dalits*. Unskilled workers, married and people with excessive alcohol use more frequently reported illnesses [63]. Physically ill patients were reported with a 31.7 % prevalence of neuropsychiatric illnesses [64].

### Injuries and accidents

Seven studies have reported on injuries and accidents. RTAs are now one of the major causes of death and disability in Nepal. On average, 3.5 % of the population had minor injuries and 0.7 % had major injuries occurring per month, reported in a study from Dharan (City in Eastern Terai region of Nepal) [65]. Mostly people in the age group 40-49 years suffered these injuries and people working with agriculture were mostly affected. Most of the minor injuries occurred at home (55.6 %) and most of the major injuries were road accidents (53.1 %) [65]. Higher prevalence of accidents and injuries was reported in males compared to females [66]. A study done in a hospital in Kathmandu reported that RTAs accounted for 6.6 % of all the cases attending the emergency department. Half of these cases belonged to the 21-40 years age group. Pedestrians (56.54 %) and motorbike riders (55.09 %) were most frequently involved in these accidents [67, 68]. Also, RTAs accounted for most of the spinal [69] and head injuries; prevalence of these injuries was higher among males compared to females (78 % vs 22 %) [68].

### Risk factors for NCDs

The Nepal Demographic Health Survey (NDHS) 2006, reported a prevalence of any tobacco use of 30.3 % (Male: 56.5 % and Female: 19.6 %) [70], which increased to 51.9 % in 2011 [71]. Percentage of cigarette smokers and smokeless forms of tobacco use was reported to be 12.9 % and 14.1 %, respectively in eastern Nepal [72]. In a college-based study in Kaski district, 13.9 % of the students had never used tobacco products; boys (20.5 %) reported a ten-fold higher rate of tobacco use than girls (2.9 %) [73].

With regard to obesity, a study from eastern Nepal reported that nearly one third (32.9 %) were overweight while 7.2 % were obese [74]. Being physically inactive was associated with higher obesity prevalence in the older population [74]. Higher prevalence of CHD risk factors such as HTN (35.3 %), diabetes mellitus (15.9 %), history of smoking (38.7 %), sedentary lifestyle (47.1 %), body mass index >25 kg/m^2^ (33.6 %), obesity (42.1 %) and hypercholesterolemia (12.6 %) was observed [75]. A study based on three consecutive NDHS surveys (1996-2006) reported the prevalence of overweight and obesity had increased by 4.2 folds with the highest rise in rural compared to urban areas. Age, increasing relative wealth and urban residence impacted positively to reduce the prevalence [76].

**Fig. 2:**
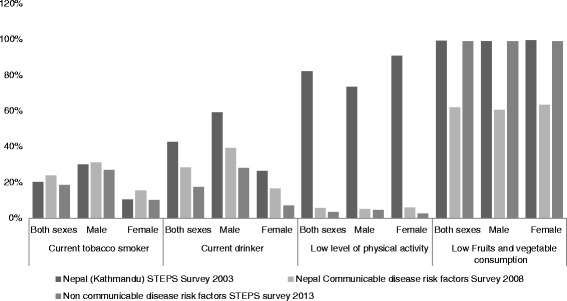
Risk factors of major NCDs reported over the consecutive STEPS surveys in Nepal

The 2003 Stepwise Approach to Surveillance (STEPS) was the first large scale study to report risk factors of NCDs in Nepal. A total of 20.1 % of the respondents were current tobacco smokers and 42.8 % of them were current alcohol consumers. Similarly, 82.3 % had a lower level of physical activity and 99.2 % had less than recommended intake of fruits and vegetables [11]. In the second STEPS survey five years later, the percentage of current tobacco smokers had decreased by 3.7 % and the percentage of current alcohol consumers had decreased by 14.3 %. This decreased further by 5.5 % and 11.1 %, respectively in 2013 compared to the levels reported in the 2008 STEPS survey [12]. Low level of physical activity was observed in 5.3 % in 2008, which slightly decreased to 3.5 % in 2013. Low fruits and vegetable consumption was found among 61.9 % in 2008, but was increased by 37.0 % in 2013. In addition, the study reported that 7.2 % were overweight/obese and 21.5 % had raised blood pressure in 2008, which was further increased to 21.6 % and 25.7 %, respectively in 2013 [13]. The 2013 STEPS survey reported that 4.1 % had impaired fasting glycaemia and 22.7 % had raised total cholesterol level [12] (Fig. 2).

## Discussion

According to earlier studies, there are currently approximately 8,000-10,000 new cancer patients per year in Nepal. Females are more affected by cancer than males [28]. Cancer of the lung, uterine cervix, head and neck, breast and gastric are the most common types of cancer in Nepal [21, 23]. Due to the high prevalence of tobacco smoking, lung cancer is the most common cancer site in both genders in Nepal [20].

A high prevalence of HTN between 22.4 % and 38.6 % has been reported [32–37]. Higher heterogenicity due to methodological differences might explain the pronounced differences in HTN prevalence. HTN prevalence is increasing with higher burdens reported in males compared to females [33, 35]. Prevalence of diabetes was found between 4.1 % to 9.5 % [9, 46–50], and prevalence of cardiovascular diseases of 5.7 % was reported [44]. Although there are methodological weaknesses in the studies of mental illness, the reported prevalence was 37.5 % [63]. This clearly shows the need for further community based studies to examine the true prevalence of mental illness.

Injuries and death related to RTAs are increasing at an alarming rate in Nepal [77]. The number of deaths of passengers and pedestrians per 10,000 vehicles has increased over the years. In 2012, RTAs were responsible for more than 1,816 deaths and an additional 13,582 people were injured; about half of these were in the Kathmandu Valley; however, the fatality rate is higher outside the Kathmandu Valley. This might be due to high growth of car traffic, mixed speed, risky driving and lack of proper roads and lighting [77]. The loss related to RTAs amounted to NRs. 22.7 billion (US$ 41.2 millions) or 0.4 % of the Gross National Product in 2007, and will incur more lives and loss of economy if adequate actions are not taken in the future.

### National response to NCDs

We have seen few global responses recently to counteract the burden of NCDs. UN high-level meeting on NCDs and the WHO Framework Convention on Tobacco Control (WHO FCTC) have laid a milestone for directing the measures to limit prevalence of NCDs or their risk factors. In line to these activities, Nepal has also made interventions to mitigate the escalating burden of NCDs. However, the proportion of government budget spent in NCD was 0.7 % in 2009. The chunk of this budget is spent only in tobacco control, nutrition and cancer programs [14]. A cancer registry system has been established in two large tertiary public hospitals. However, registries are still not available in the other public and private hospitals. While information resources and advocacy on harmful effects of tobacco products are abundant, the same does not apply to other modifiable risk factors of NCDs. Smoking in public places was banned in early 1992. In 1999, the government banned advertisements for alcohol in electronic media. Following that, in 2001, the government introduced specific provisions for sale, distribution and consumption of alcohol. Also, Nepal ratified the WHO FCTC following that the Tobacco Products (control and regulatory) act was developed in 2011. This policy is currently being incorporated with interventions such as greater graphic health warnings on all tobacco products. The Animal Health and Livestock Service Act from 1998 and its regulations in 1999, the Pesticide Act from 1991 and its regulations in 1993 came into the limelight, including Department of Food Technology and Quality Control to monitor food safety. The government has drafted the Mental Health Act 2012, prepared in line with several international human rights conventions. Moreover, the government-owned Nepal Health Sector Program II focuses on developing and scaling up mental health projects and training health cadres at district level. In light of the increasing number of RTAs, a Road Safety Action Plan (2012-2020) is being implemented [78]. The country already made an NCD policy and strategy in 2009 [15] but it has not been implemented yet. Following the high level political declaration on NCDs, the government of Nepal also formed a high level steering committee on NCDs and the multi-sectoral plan on NCDs was prepared in 2013 [78] (Table 2). This plan is waiting to be implemented.

**Table 2:** Eleven strategic government policies for NCD management, adapted from Multisectoral Action Plan on the Prevention and Control of NCDs in Nepal 2014-2020 [78]

Strategic policies	Action points	Targets
High level political commitment	Action area 1: Leadership, advocacy and partnership	1. 25 % relative reduction in overall mortality from cardiovascular diseases, cancers, diabetes, or chronic respiratory diseases
To have high level of political commitment in line with country international commitment, NCD multisectoral action plan will be linked to the head of state/his representative Chief Secretary Government of Nepal	• Establishment of National Steering Committee for NCD Prevention And Control chaired by Chief Secretary,	2. 10 % relative reduction in the harmful use of alcohol
Multisectoral response	• Creation of functional NCD Unit at the MoHP to coordinate NCD activities	3. 30 % relative reduction in prevalence of current tobacco use in persons over 15 years
Accelerating and scaling up national response to NCD epidemic by setting functional mechanism for multisectoral partnerships and effective coordination, effective leadership and sustained political commitment and resources for implementation of NCD action plan	• Encouraging formation of regional and district NCD committees to oversee activities at each level, numerous inter-sectoral planning and	4. 50 % relative reduction in the proportion of households using solid fuels as the primary source of cooking
Tobacco	• Encouraging review of work plans and sharing lessons of implementation.	5. 30 % relative reduction in mean population intake of salt/sodium
Strengthening enforcement and compliance to Tobacco Product (control and regulatory) Act, 2011 and improving public awareness to hazards of tobacco use	Action area 2: Health promotion and risk reduction.	6. 25 % reduction in prevalence of raised blood pressure
Alcohol	• Enforcement of the existing tobacco regulations, encourage implementation of alcohol policies in line with the Global policy	7. Halt the rise in obesity and diabetes
Reducing commercial and public availability of alcohol and implementing social mobilisation programmes to reduce harmful use of alcohol	• Strategy to reduce harmful use of alcohol	8. 10 % relative reduction in prevalence of insufficient physical activity
Unhealthy diet	• Encourage increased consumption of fruits and vegetables and legislate ban of food products with high unsaturated fat and reduce salt consumption	9. 50 % of eligible people receive drug therapy and counseling (including glycemic control) to prevent heart attacks and strokes
Encouraging increased consumption of fruits and vegetables, reducing consumption of salt, saturated fat and unsaturated fat	• Community-based projects to reduce indoor air pollution will be scaled up in rural communities.	10. 80 % availability of affordable basic technologies and essential medicines, including generics, required to treat major NCDs in both public and private facilities
Physical inactivity	Action area 3: Health systems strengthening for Early detection and management of NCDs and their risk factors.	
Improving environment and promoting health beneficial physical activity through supportive policies in key settings	• Strengthening the existing primary health care system through a Package of Essential NCDs (PENs) will be piloted in primary health care settings. Essential drug lists will be updated with psychotropic drugs, diagnostic services for NCDs will be added, primary health care workers will be trained in NCD management and referral system will be developed.	
Indoor air pollution	Action area 4: Surveillance, monitoring and evaluation, and research.	
Reaching communities and areas with poor indoor air quality as a result of use of biomass fuels for cooking and heating, and providing support with alternative means of energy to reduce adverse health impacts	• Surveys in mental health, oral diseases, sodium urinary excretion level, national psychiatric morbidity survey, assessment of fluoride content in water and assessment of physical infrastructure for walk-ability in urban settings will be conducted.	
Essential NCDs (CVDs, COPDs, diabetes and cancer)		
Strengthening health system competence, particularly the primary health care system to address common NCDs particularly CVDs, COPDs, diabetes and cancer, along with the additional NCDs and empowering communities and individuals to perform self-care		
Oral health		
Improving access to essential oral health services through community oriented oral health focusing on preventable oral diseases and oral care		
Mental health		
Improving basic minimum care of mental health services in the community and improving competency for case identification and initiating referral at primary care level		
Surveillance, research, monitoring and evaluation		
Strengthening systematic data collection on NCDs and their risk factors, programme implementation and use of this information for evidence-based policy and programme development		

While the country has a burgeoning burden of NCDs compared to any other diseases, it is at the very first stage in responding to NCDs. The primary health care system does not provide NCD-related services. There are some public facilities for kidney transplantation, cancer and cardiovascular care, but they are mostly focused on curative treatment. Rural areas do not have preventive and curative services for NCDs as health care is concentrated around the cities. Greater emphasis should be given to implementation of the NCD action plan with particular focus on providing preventive and curative NCD care and conducting periodic operational studies and surveys to map out the burden of NCDs in Nepal.

## Conclusions

There are limitations in available studies in this review including generalisability due to small sample sizes, non-random sampling and lack of studies from certain region of country. Limited studies on population-based prevalence of mental illness, chronic respiratory diseases, cardiovascular diseases, and road traffic accidents were found. Overall, the most common site of cancer in males was the lung, followed by the oral cavity and gastric, while the most prevalent three in females were cervix uteri, breast and lung. Females are more affected by cancer than males. The reported prevalence of hypertension was 22.4 %-38.6 %. The prevalence of diabetes was reported between 4.1 % and 9.5 %. Prevalence of CVD of 5.7 % was reported. Prevalence of mental illness cases was 37.5 %. Chronic respiratory diseases constitute a considerable outpatient burden (31.7 %), most frequent diagnosis is COPD (23.2%). Risk factors of NCDs were high levels of tobacco use (51.9 %), current alcohol intake (17.4%), overweight (32.9 %), low physical activity (3.5 %), and low vegetable and fruits consumption (98.9%). However, the country's response to NCDs is weak with inadequate preventive and curative services at public and private level. Nepal needs to build non-communicable disease programmes and implement already developed NCD control plans.

## Additional file

Additional file 1:
**Single/combined search terms used in Pubmed/Medline.**

## References

[1] World Health Organization. Global status report on noncommunicable diseases 2014. Geneva, Switzerland: WHO; 2014.

[2] Prentice AM (2006). The emerging epidemic of obesity in developing countries. Int J Epidemiol.

[3] World Health Organization. Preventing chronic diseases: a vital investment: WHO global report. Geneva, Switzerland: WHO; 2005.

[4] Moodie R, Swinburn B, Richardson J, Somaini B (2006). Childhood obesity--a sign of commercial success, but a market failure. Int J Pediatr Obes.

[5] Kennedy G, Nantel G, Shetty P. Globalization of food systems in developing countries: a synthesis of country case studies. Food and Nutrition Paper 83. Rome, Italy: FAO; 2004. 19178111

[6] van Deurzen I, van Oorschot W, van Ingen E (2014). The link between inequality and population health in low and middle income countries: policy myth or social reality?. PLoS One.

[7] World Health Organization. World Health Statistics 2010. Geneva, Switzerland; 2010.

[8] Neupane D, McLachlan CS, Sharma R, Gyawali B, Khanal V, Mishra SR (2014). Prevalence of hypertension in member countries of South Asian Association for Regional Cooperation (SAARC): systematic review and meta-analysis. Medicine.

[9] Singh DL, Bhattarai MD (2003). High prevalence of diabetes and impaired fasting glycaemia in urban Nepal. Diabet Med.

[10] Bhandari GP, Angdembe MR, Dhimal M, Neupane S, Bhusal C (2014). State of non-communicable diseases in Nepal. BMC Public Health.

[11] World Health Organization (2003). Research Report on NCD Risk Factors Surveillance in Nepal.

[12] Aryal KK, Neupane S, Mehata S, Vaidya A, Singh S, Paulin F (2014). Non communicable diseases risk factors: STEPS Survey Nepal 2013.

[13] Ministry of Health and Population, Government of Nepal, Society for Local Integrated Development Nepal, WHO (2008). WHO STEPS Surveillance: Non Communicable Disease Risk Factors Survey.

[14] World Bank. Non-Communicable Disease in Nepal-Nepal's Next Major Health Challenge In: NCDs Policy Brief-Nepal. Washington DC, USA: World Bank; 2011.

[15] Ministry of Health and Population(MOHP) [Nepal] (2009). Integrated non-communicable diseases(NCDs) prevention and control policy of Nepal.

[16] Gautam R. NCDs in Nepal: burgeoning burden amid low priority and the ways forward. Health Prospect. 2013, 11:iv-v.

[17] Arksey H, O'Malley L (2005). Scoping studies: towards a methodological framework. Int J Soc Res Methodol.

[18] Pham MT, Rajić A, Greig JD, Sargeant JM, Papadopoulos A, McEwen SA. A scoping review of scoping reviews: advancing the approach and enhancing the consistency. Res Synth Methods. 2014;5(4):371–85. 10.1002/jrsm.1123 PMC4491356 26052958

[19] Schwartz J, Guwatudde D, Nugent R, Kiiza C (2014). Looking at non-communicable diseases in Uganda through a local lens: an analysis using locally derived data. Global Health.

[20] Subedi K, Sharma P (2012). Cancer treatment in Nepal: a historical background, development of treatment facilities, epidemiology and challenges for prevention and control of cancer. Austr-Asian J Canc.

[21] Pradhananga KK, Baral M, Shrestha BM (2009). Multi-institution hospital-based cancer incidence data for Nepal: an initial report. Asian Pac J Cancer Prev.

[22] Khan GM, Thapa RK, Adhikari DS, Rajbhandari M, Dwa P, Shrestha S (2013). Evaluation of cancer prevalence and cytotoxic medication prescribing in central region of Nepal. Kathmandu Univ J Sci Eng Technol.

[23] Ghimire B, Singh YP, Timalsina S (2014). Post operative diagnosis of early gastric cancer in a low risk population and the possibility of risk stratified screening. Kathmandu Univ Med J (KUMJ).

[24] Bajracharya N, Karki P, Sapkota S, Bastakoti S, Yagol N, Khan G, et al. Prevalence pattern of cancer and handling of cytotoxic drugs. Kathmandu Univ J Sci Eng Technol. 2006;2(1):1–7.

[25] Sherpa AT, Clifford GM, Vaccarella S, Shrestha S, Nygard M, Karki BS (2010). Human papillomavirus infection in women with and without cervical cancer in Nepal. Cancer Causes Control.

[26] Johnson DC, Bhatta MP, Smith JS, Kempf MC, Broker TR, Vermund SH (2014). Assessment of high-risk human papillomavirus infections using clinician- and self-collected cervical sampling methods in rural women from far western Nepal. PLoS One.

[27] Jha AK, Jha J, Bista R, Basnet B, Kandel P, Lama G (2009). A scenario of cervical carcinoma in a cancer hospital. JNMA J Nepal Med Assoc.

[28] Khan GM, Thappa R, Adhikari D, Rajbhandari M, Dwa P, Shrestha S, et al. Cancer Prevalence Trend in Central Region of Nepal. Journal of Chitwan Medical College. vol. 3; 2013.

[29] Shrestha UK, Ghosh A, Alurkar VM, Kohli SC, Sapkota S (2013). Prevalence of Helicobacter pylori infection, its correlation with gastroduodenal diseases and the incidence of gastric cancer in Nepal. J Adv Int Med.

[30] Shrestha M, Shah T. Prevalence of breast lump and risk factors of breast cancer among reproductive aged women of Jabalpur VDC of Sunsari District, Nepal. J Nepal Health Res Counc. 2004;2(1):1–4.

[31] Chobanian AV, Bakris GL, Black HR, Cushman WC, Green LA, Izzo JL (2004). The seventh report of the joint national committee on prevention, detection, evaluation, and treatment of high blood pressure: the JNC 7 report. JAMA.

[32] Sharma SK, Ghimire A, Radhakrishnan J, Thapa L, Shrestha NR, Paudel N (2011). Prevalence of hypertension, obesity, diabetes, and metabolic syndrome in Nepal. Int J Hypertens.

[33] Vaidya A, Pathak RP, Pandey MR (2012). Prevalence of hypertension in Nepalese community triples in 25 years: a repeat cross-sectional study in rural Kathmandu. Indian Heart J.

[34] Stewart CP, Christian P, Wu LS, LeClerq SC, Khatry SK, West KP (2013). Prevalence and risk factors of elevated blood pressure, overweight, and dyslipidemia in adolescent and young adults in rural Nepal. Metab Syndr Relat Disord.

[35] Chataut J, Adhikari RK, Sinha NP (2011). The prevalence of and risk factors for hypertension in adults living in central development region of Nepal. Kathmandu Univ Med J (KUMJ).

[36] Sharma SK, Dhakal S, Thapa L, Ghimire A, Tamrakar R, Chaudhary S (2013). Community-based screening for chronic kidney disease, hypertension and diabetes in Dharan. JNMA J Nepal Med Assoc.

[37] Vaidya A, Pokharel PK, Karki P, Nagesh S (2007). Exploring the iceberg of hypertension: a community based study in an eastern Nepal town. Kathmandu Univ Med J (KUMJ).

[38] Koju R, Manandhar K, Gurung R, Pant P, Bedi T (2013). Prevalence of Hypertension in Semi-urban area of Nepal. Nepalese Heart J.

[39] Shrestha UK, Singh DL, Bhattarai MD (2006). The prevalence of hypertension and diabetes defined by fasting and 2-h plasma glucose criteria in urban Nepal. Diabet Med.

[40] Khan RJ, Stewart CP, Christian P, Schulze KJ, Wu L, Leclerq SC (2013). A cross-sectional study of the prevalence and risk factors for hypertension in rural Nepali women. BMC Public Health.

[41] Vaidya A, Pokharel PK, Nagesh S, Karki P, Kumar S, Majhi S (2007). War veterans of Nepal and their blood pressure status: a population-based comparative study. J Hum Hypertens.

[42] Manandhar K, Koju R, Sinha NP, Humagain S (2012). Prevalence and associated risk factors of hypertension among people aged 50 years and more in Banepa Municipality, Nepal. Kathmandu Univ Med J (KUMJ).

[43] RJ Khan, CP Stewart, P Christian, KJ Schulze, L Wu, SC LeClerq, et al. A cross-sectional study of the prevalence and risk factors for hypertension in rural Nepali women. BMC Public Health. 2013, 13(55). 10.1186/1471-2458-13-55 PMC3566953 23336578

[44] Vaidya A, Pokharel PK, Nagesh S, Karki P, Kumar S, Majhi S (2009). Prevalence of coronary heart disease in the urban adult males of eastern Nepal: a population-based analytical cross-sectional study. Indian Heart J.

[45] Maskey A, Sayami A, Pandey M (2003). Coronary artery disease: an emerging epidemic in Nepal. J Nepal Med Ass.

[46] Baral N, Koner BC, Karki P, Ramaprasad C, Lamsal M, Koirala S (2000). Evaluation of new WHO diagnostic criteria for diabetes on the prevalence of abnormal glucose tolerance in a heterogeneous Nepali population--the implications of measuring glycated hemoglobin. Singapore Med J.

[47] Pokharel D, Gautam N, Archana J, Nagamma T, Kumar R, Sapkota RM. Frequency of Type 2 Diabetes mellitus and Impaired Glycemia in a Teaching Hospital of South-Western Nepal. Asian J Med Sci. vol. 2; 2012.

[48] Karki P, Baral N, Lamsal M, Rijal S, Koner BC, Dhungel S (2000). Prevalence of non-insulin dependent diabetes mellitus in urban areas of eastern Nepal: a hospital based study. Southeast Asian J Trop Med Public Health.

[49] Ono K, Limbu YR, Rai SK, Kurokawa M, Yanagida J, Rai G (2007). The prevalence of type 2 diabetes mellitus and impaired fasting glucose in semi-urban population of Nepal. Nepal Med Coll J.

[50] Paudyal G, Shrestha MK, Meyer JJ, Thapa R, Gurung R, Ruit S (2008). Prevalence of diabetic retinopathy following a community screening for diabetes. Nepal Med Coll J.

[51] Alberti KG, Zimmet PZ (1998). Definition, diagnosis and classification of diabetes mellitus and its complications. Part 1: diagnosis and classification of diabetes mellitus provisional report of a WHO consultation. Diabet Med.

[52] Riccardi G, Vaccaro O, Rivellese A, Pignalosa S, Tutino L, Mancini M (1985). Reproducibility of the new diagnostic criteria for impaired glucose tolerance. Am J Epidemiol.

[53] Mandal F, Gupta S, Rimal B, Kafle D. Prevalence of gestational diabetes mellitus in National Medical College & Teaching Hospital, Birgunj, Nepal. Int Res J Pharm App Sci. 25(35):25-35.

[54] Mehta KD, Karki P, Lamsal M, Paudel IS, Majhi S, Das BK (2011). Hyperglycemia, glucose intolerance, hypertension and socioeconomic position in eastern Nepal. Southeast Asian J Trop Med Public Health.

[55] Sasaki H, Kawasaki T, Ogaki T, Kobayashi S, Itoh K, Yoshimizu Y (2005). The prevalence of diabetes mellitus and impaired fasting glucose/glycaemia (IFG) in suburban and rural Nepal-the communities--based cross-sectional study during the democratic movements in 1990. Diabetes Res Clin Pract.

[56] Rimal A, Panza A. Prevalence of, and factors associated with, type 2 diabetes and its microvascular complications among the elderly in Kathmandu, Nepal. J Health Res. 2013;27(1):45–9.

[57] Fossen T, Nossen J (2013). The prevalence of diabetes mellitus and associated risk factors in the female population of Kavre in rural Nepal.

[58] Chhetri MR, Chapman RS (2009). Prevalence and determinants of diabetes among the elderly population in the Kathmandu Valley of Nepal. Nepal Med Coll J.

[59] Pokharel B, Humagain S, Pant P, Gurung R, Koju R, Bedi T. Spectrum of diseases in a medical ward of a teaching hospital in a developing country. J Coll Med Sci Nepal. vol. 8; 2012.

[60] Pandey MR (1984). Prevalence of chronic bronchitis in a rural community of the Hill Region of Nepal. Thorax.

[61] Bhandari R, Sharma R (2012). Epidemiology of chronic obstructive pulmonary disease: a descriptive study in the mid-western region of Nepal. Int J Chron Obstruct Pulmon Dis.

[62] Dhungel S, Paudel B, Shah S (2005). Study of prevalence of hypertension in Chronic Obstructive Pulmonary Disease patients admitted at Nepal Medical College and Teaching Hospital. Nepal Med Coll J.

[63] Khattri J, Poudel B, Thapa P, Godar S, Tirkey S, Ramesh K, et al. An Epidemiological Study of Psychiatric Cases in a Rural Community of Nepal. NJMS. 2013;2(1):52–56.

[64] Shyangwa PM, Joshi D, Sherchan S, Thapa KB (2009). Psychiatric morbidity among physically ill persons in eastern Nepal. Nepal Med Coll J.

[65] Ghimire A, Nagesh S, Jha N, Niraula SR, Devkota S (2009). An epidemiological study of injury among urban population. Kathmandu Univ Med J (KUMJ).

[66] Rajbhandari S. Prevalence of Injuries amongst the People of Sonapur Village Development Committee Eastern Nepal. Int J Pharm & Biol Arch. 2013;4(1):56–60.

[67] Banthia P, Koirala B, Rauniyar A, Chaudhary D, Kharel T, Khadka SB (2006). An epidemiological study of road traffic accident cases attending emergency department of teaching hospital. JNMA J Nepal Med Assoc.

[68] Jha S, Yadav B, Karn A, Aggrawal A, Gautam A. Epidemiological Study of Fatal Head Injury in Road Traffic Accident Cases: A Study from BPKIHS, Dharan. Health Renaissance. 2010;8(2):97–101.

[69] Lakhey S, Jha N, Shrestha BP, Niraula S (2005). Aetioepidemiological profile of spinal injury patients in Eastern Nepal. Trop Doct.

[70] Sreeramareddy CT, Ramakrishnareddy N, Harsha Kumar H, Sathian B, Arokiasamy JT (2011). Prevalence, distribution and correlates of tobacco smoking and chewing in Nepal: a secondary data analysis of Nepal Demographic and Health Survey-2006. Subst Abuse Treat Prev Policy.

[71] Khanal V, Adhikari M, Karki S (2013). Social determinants of tobacco consumption among Nepalese men: findings from Nepal Demographic and Health Survey 2011. Harm Reduct J.

[72] Niraula SR (2004). Tobacco use among women in Dharan, eastern Nepal. J Health Popul Nutr.

[73] Sreeramareddy CT, Kishore P, Paudel J, Menezes RG (2008). Prevalence and correlates of tobacco use amongst junior collegiates in twin cities of western Nepal: a cross-sectional, questionnaire-based survey. BMC Public Health.

[74] Vaidya AK, Pokharel PK, Nagesh S, Karki P, Kumar S, Majhi S (2006). Association of obesity and physical activity in adult males of Dharan, Nepal. Kathmandu Univ Med J (KUMJ).

[75] Kalra S, Narain S, Karki P, Ansari JA, Ranabhat K, Basnet N (2011). Prevalence of risk factors for coronary artery disease in the community in eastern Nepal--a pilot study. J Assoc Physicians India.

[76] Balarajan Y, Villamor E (2009). Nationally representative surveys show recent increases in the prevalence of overweight and obesity among women of reproductive age in Bangladesh, Nepal, and India. J Nutr.

[77] Thapa AJ. Status Paper on Road Safety in Nepal. DDG, Department of Roads. Kathmandu, Nepal: 2013.

[78] Multisectoral Action Plan on the Prevention and Control of NCD in Nepal 2014-2020 [http://www.searo.who.int/nepal/mediacentre/ncd_multisectoral_action_plan.pdf]

