# Supplementary material for: Burgeoning burden of non-communicable diseases in Nepal: a scoping review
Source: Global Health. 2015 Jul 16;11:32. doi: 10.1186/s12992-015-0119-7 (PMC4504073; doi:10.1186/s12992-015-0119-7)
Supplement: Additional file 1: — Single/combined search terms used in Pubmed/Medline. [file 12992_2015_119_MOESM1_ESM.docx]

**Supplementary material 1: Single/combined search terms used in Pubmed/Medline**

("Nepal"[Mesh] AND "Neoplasms"[Mesh] AND "Prevalence"[Mesh]) OR ("Nepal"[Mesh] AND "Hypertension"[Mesh] AND "Prevalence"[Mesh]) OR ("Nepal"[Mesh] AND " Diabetes Mellitus"[Mesh] AND "Prevalence"[Mesh]) OR ("Nepal"[Mesh] AND " Hyperglycemia "[Mesh] AND "Prevalence"[Mesh]) OR ("Nepal"[Mesh] AND " Obesity"[Mesh] AND "Prevalence"[Mesh]) OR ("Nepal"[Mesh] AND " Kidney"[Mesh] AND "Prevalence"[Mesh]) OR ("Nepal"[Mesh] AND " Liver "[Mesh] AND "Prevalence"[Mesh]) OR ("Nepal"[Mesh] AND " Wounds and Injuries "[Mesh] AND "Prevalence"[Mesh]) OR ("Nepal"[Mesh] AND " Accidents, Traffic"[Mesh] AND "Prevalence"[Mesh]) OR ("Nepal"[Mesh] AND " Tobacco Products "[Mesh] AND "Prevalence"[Mesh]) OR ("Nepal"[Mesh] AND " Alcohols "[Mesh] AND "Prevalence"[Mesh]) OR ("Nepal"[Mesh] AND " Exercise "[Mesh] AND "Prevalence"[Mesh]) OR ("Nepal"[Mesh] AND " Vegetables "[Mesh] AND "Prevalence"[Mesh])
